# Factors associated with receptive injection equipment sharing among people who inject drugs: findings from a multistate study at the start of the COVID-19 pandemic

**DOI:** 10.1186/s12954-023-00746-5

**Published:** 2023-02-15

**Authors:** Sean T. Allen, Kristin E. Schneider, Miles Morris, Saba Rouhani, Samantha J. Harris, Brendan Saloner, Susan G. Sherman

**Affiliations:** 1 Department of Health, Behavior, Society; Bloomberg School of Public Health, Johns Hopkins University, 624 N. Broadway, Hampton House 184, Baltimore, MD 21205 USA; 2 Department of Health Policy and Management; Bloomberg School of Public Health, Johns Hopkins University, 624 N. Broadway, Baltimore, MD 21205 USA

**Keywords:** Injection drug use, Injection equipment sharing, People who inject drugs, COVID-19

## Abstract

**Background:**

Receptive injection equipment sharing (i.e., injecting with syringes, cookers, rinse water previously used by another person) plays a central role in the transmission of infectious diseases (e.g., HIV, viral hepatitis) among people who inject drugs. Better understanding these behaviors in the context of COVID-19 may afford insights about potential intervention opportunities in future health crises.

**Objective:**

This study examines factors associated with receptive injection equipment sharing among people who inject drugs in the context of COVID-19.

**Methods:**

From August 2020 to January 2021, people who inject drugs were recruited from 22 substance use disorder treatment programs and harm reduction service providers in nine states and the District of Columbia to complete a survey that ascertained how the COVID-19 pandemic affected substance use behaviors. We used logistic regression to identify factors associated with people who inject drugs having recently engaged in receptive injection equipment sharing.

**Results:**

One in four people who inject drugs in our sample reported having engaged in receptive injection equipment sharing in the past month. Factors associated with greater odds of receptive injection equipment sharing included: having a high school education or equivalent (adjusted odds ratio [aOR] = 2.14, 95% confidence interval [95% CI] 1.24, 3.69), experiencing hunger at least weekly (aOR = 1.89, 95% CI 1.01, 3.56), and number of drugs injected (aOR = 1.15, 95% CI 1.02, 1.30). Older age (aOR = 0.97, 95% CI 0.94, 1.00) and living in a non-metropolitan area (aOR = 0.43, 95% CI 0.18, 1.02) were marginally associated with decreased odds of receptive injection equipment sharing.

**Conclusions:**

Receptive injection equipment sharing was relatively common among our sample during the early months of the COVID-19 pandemic. Our findings contribute to existing literature that examines receptive injection equipment sharing by demonstrating that this behavior was associated with factors identified in similar research that occurred before COVID. Eliminating high-risk injection practices among people who inject drugs requires investments in low-threshold and evidence-based services that ensure persons have access to sterile injection equipment.

## Background

High-risk injection practices, such as receptive injection equipment sharing (i.e., injecting with syringes, cookers, rinse water that were previously used by another person), play a central role in the transmission of infectious diseases (e.g., HIV, viral hepatitis) among people who inject drugs (PWID) [1–4]. In the United States (US), there are an estimated 750,000 people who injected drugs in the past year [5]. Studies have found that the prevalence of receptive injection equipment sharing among PWID varies across the United States and has been associated with infectious disease outbreaks [6–19]. For example, a study conducted in Baltimore City (Maryland) found that 16% of PWID reported having engaged in receptive syringe sharing in the past month [7]. Another study conducted among PWID in a rural county in West Virginia found that 43% reported engaging in receptive syringe sharing in the past 6 months [9]. Similarly, a study conducted in Kentucky found that 30.2% of a sample of PWID living with viral hepatitis reported having recently engaged in receptive syringe sharing [4]. These and other findings underscore the continued need for comprehensive interventions that increase access to sterile injection equipment.

Several decades of research have been conducted to better understand unsafe injection practices among PWID. For example, prior studies have identified that these behaviors are driven by the intersections of individual- and structural-level factors, substance use, social context, and policy [7, 9, 13, 16]. Inadequate access to sterile injection equipment has also been associated with syringe sharing [20–22]. Mitigating the consequences of high-risk injection practices (e.g., infectious disease acquisition) may be achieved through the implementation of interventions that aim to increase access to sterile injection equipment, including syringe services programs (SSPs) [23–26]. However, many communities lack SSPs due to restrictive policies, community-level opposition, and inaccurate fears that they may increase substance use, crime, or syringe litter [20, 26–35]. Stigma and discrimination against people who use drugs also negatively affect the implementation and utilization of SSPs and other evidence-based response strategies, such as medications for opioid use disorder (MOUD).

The COVID-19 pandemic had far-reaching effects on public health, including among PWID. In some instances, SSPs closed or modified their operations to reduce COVID-19 transmission risks [36–40]. Some SSPs also had inadequate staffing during the pandemic which led to decreased service availability, such as onsite HIV and hepatitis testing [36]. Further, pandemic lockdowns also resulted in reductions in syringe distribution and infectious disease testing [41]. Mental health issues (e.g., depression, anxiety, and loneliness) worsened among people who use drugs during the pandemic [37, 42, 43]. In terms of substance use disorder treatment, a 2022 study found that there were substantial reductions in in-person services, but policy changes that provided flexibilities in treatment delivery (e.g., increased take-home medications, counseling by video/phone, and fewer urine drug screens) were well-received among people with histories of substance use [44]. Other COVID-19 era research has found that PWID struggled to get appointments with HIV counselors and physicians and that access to preexposure prophylaxis diminished during the pandemic [45, 46].

Although existing research demonstrates several ways in which the COVID-19 pandemic affected PWID, limited research has been conducted to understand its impact on high-risk injection practices. One study found that syringe reuse was more common during the pandemic [43], but this was limited to a sample of PWID in New York City and may not be generalizable to other settings. Given that receptive injection equipment sharing is strongly associated with infectious disease transmission among PWID, better understanding this behavior in the context of COVID-19 may afford key insights about potential intervention opportunities in the ongoing pandemic and in ensuring sustainable access to sterile supplies in the future. This study utilizes data from a multistate survey conducted in late 2020 and early 2021 to examine factors associated with receptive injection equipment sharing among PWID.

## Methods

### Study context

From August 2020 to January 2021, study participants were recruited from 22 substance use disorder treatment programs and harm reduction service providers in nine states (Maine, Maryland, Michigan, New Jersey, New Mexico, New York, Pennsylvania, Tennessee, and West Virginia) and the District of Columbia. Most participating drug treatment programs and harm reduction providers were engaged in the Bloomberg Opioid Initiative (a campaign supported by Bloomberg Philanthropies that aims to reduce overdose rates). Staff at collaborating organizations distributed study recruitment cards to clients. Each card featured the study logo, the study phone number, and a unique study identifier (to reduce duplicate and non-client participation). Persons who were interested in participating in the study contacted the data collection team via phone and were subsequently able to ask questions and be screened for eligibility. Eligibility criteria included being at least 18 years old, a current client of a collaborating organization, able to provide informed consent, and able to provide an unused unique study identifier. Participants received $40 compensation via a pre-paid gift card or Venmo payment. Overall, 587 responses were collected. Given our interest in receptive injection equipment sharing among PWID, we restricted the analytic sample to participants who had injected drugs in the past month (*n* = 266). We further removed a transgender participant to ensure their anonymity was protected. This research was approved by the Johns Hopkins School of Public Health Institutional Review Board.

### Measures

#### Receptive injection equipment sharing in the past month

Participants answered two questions about their receptive injection equipment sharing behaviors in the past month. Participants indicated if they had used a syringe or needle after someone else had used it and if they had used other injection equipment, like cookers or rinse water, after someone else. These two indicators had a high degree of overlap (85% of persons who shared syringes also shared other equipment); as a result, we created a binary indicator for receptive sharing of any injection equipment in the past month.

#### Sociodemographic characteristics

Participants reported their age (in years), gender (man/woman), relationship status (single/in a relationship or married), sexual orientation (heterosexual or straight/sexual minority), education level (less than high school, high school diploma or equivalent, or some college or more), and employment status (full time, part time, not working). Participants reported their race and ethnicity, which we dichotomized to non-Hispanic White and Racial/Ethnic Minority (e.g., Black, Hispanic, Multiracial/Multiethnic) due to sample size constraints. Participants further reported if they were currently homeless (yes/no), if they experienced hunger (defined as going to bed hungry due to lack of food) at least once a week since the COVID-19 pandemic (yes/no), if they had ever tested positive for HIV (yes/no), and if they traded sex for drugs or money since the pandemic started (yes/no). Based on the county participants reported living in, we created an urbanicity measure using the National Center for Health Statistics Rural Classification Scheme (codes range from 1– large central metro to 6 – non-core). We created a three-category measure of urbanicity: large metropolitan (codes 1 and 2), small metropolitan (codes 3 and 4), and non-metropolitan (codes 5 and 6).

#### Injection drug use in the past month

We created binary indicators of whether participants reported having injected each of the following drugs/combinations of drugs in the past month: cocaine, heroin, fentanyl, heroin and fentanyl simultaneously, speedball (cocaine and heroin simultaneously), methamphetamine, methamphetamine and heroin simultaneously, prescription opioids, tranquilizers, and buprenorphine (e.g., Suboxone). We also created a variable that reflected the total number of drugs/combinations of drugs injected in the past month.

#### COVID-related drug use behavior changes

We included four measures of drug use-related behavior changes during COVID-19. First, we asked participants to indicate how often they injected drugs per day during COVID-19 relative to the pre-COVID era (less frequently, the same, more frequently). Participants indicated how often they used drugs with others during COVID-19 relative to before the pandemic (less frequently, the same, more frequently). Participants further indicated if they used mostly in private locations during COVID-19 (yes/no) and if they had avoided accessing syringe services programs due to COVID-19 fears (yes/no).

#### Service utilization

We included three binary measures of drug treatment engagement. First, we created an indicator for any past-month drug treatment. We then created two indicators for the type of treatment received: any MOUD (buprenorphine, methadone, or naltrexone) and any non-MOUD treatment. The treatment types were not mutually exclusive. We also asked participants whether they had acquired sterile syringes from a syringe services program in the past month (yes/no).

### Analysis

We first estimated the prevalence of past month receptive injection equipment sharing in our sample. We used Chi Square and t-tests, as appropriate, to assess bivariate relationships between variables and receptive injection equipment sharing. We used logistic regression to identify factors associated with PWID having recently engaged in receptive injection equipment sharing. We considered all correlates of receptive injection equipment sharing at the *p* < 0.2 level for inclusion in multivariable logistic regression analyses. We elected to utilize the number of drugs injected instead of individual drug measures to achieve a more parsimonious model. We further excluded two variables (homelessness and MOUD treatment) from the multivariable model due to collinearity with other included variables (hunger and any drug treatment, respectively). In the multivariable logistic regression model, standard errors were clustered by the provider participants were recruited from to account for study design. Statistical analyses were performed using Stata 17 (StataCorp, College Station, TX).

## Results

The average age of the sample was 39 years old (SD: 10.5). Half (50.2%) the participants were women and 62.9% identified as non-Hispanic White (Table 1). Fourteen percent identified as a sexual minority. Few (4.9%) reported having HIV. Over half (56.8%) of participants were in a relationship. Having a high school education was the most common education level (45.7%); the prevalence of having less than a high school education (27.2%) or some college or more (27.2%) were similar. Most (85.3%) participants were not working. About one-quarter (27.7%) of participants were homeless and one-third (34.3%) reported weekly hunger. Urbanicity level varied (39.0% large metropolitan, 37.5% small metropolitan, 23.5% non-metropolitan). Approximately eleven percent (10.6%) reported engaging in transactional sex. On average, participants reported injecting three drugs in the past month. Most (85.6%) had accessed an SSP in the past month. One-third (32.1%) of participants reported more frequent drug injection during COVID-19. Just under half (46.0%) had received drug treatment in the past month. One in four participants reported having engaged in receptive injection equipment sharing in the past month.

**Table 1:** Sample characteristics and correlates of receptive injection equipment sharing among PWID in the United States (*N* = 265)

	Total *N* (%)	Receptive sharing, past month		
		No *N* (%)	Yes *N* (%)	*p*
	*n* = 265	198 (74.7)	67 (25.3)	–
*Sociodemographic characteristics*				
Age, M (SD)	39.3 (10.5)	40.1 (10.8)	37.1 (9.3)	**0.04**
Gender				
Man/male	132 (49.8)	94 (47.5)	39 (58.2)	0.13
Women/female	133 (50.2)	104 (52.5)	28 (41.8)	
Sexual minority	38 (14.3)	23 (11.6)	15 (22.4)	**0.03**
Non-Hispanic, White	166 (62.9)	114 (57.9)	52 (77.6)	**0.004**
Has HIV	13 (4.9)	10 (5.1)	3 (4.5)	0.85
Single relationship status	114 (43.2)	89 (45.2)	25 (37.3)	0.26
Education				
Less than high school diploma	72 (27.2)	60 (30.3)	12 (17.9)	**0.01**
High school diploma or equivalent	121 (45.7)	80 (40.4)	41 (61.2)	
Some college or more	72 (27.2)	58 (29.3)	14 (20.9)	
Employment				
Full time	14 (5.3)	11 (5.6)	3 (4.5)	0.90
Part time	25 (9.4)	18 (9.1)	7 (10.5)	
Not working	226 (85.3)	169 (85.4)	57 (85.1)	
Homeless	73 (27.7)	50 (25.4)	23 (34.3)	0.16
Weekly hunger	91 (34.3)	61 (10.8)	30 (44.8)	**0.04**
Urbanicity categories				
Large Metropolitan area	103 (39.0)	76 (38.6)	27 (40.3)	0.13
Small Metropolitan area	99 (37.5)	69 (35.0)	30 (44.8)	
Non-Metropolitan area	62 (23.5)	52 (26.4)	10 (14.9)	
Transactional sex	28 (10.6)	16 (8.1)	12 (17.9)	**0.02**
*Past month injection drug use*				
Cocaine	38 (14.3)	26 (13.1)	12 (17.9)	0.34
Heroin	215 (81.4)	160 (81.2)	55 (82.1)	0.87
Fentanyl	112 (42.3)	82 (41.4)	30 (44.8)	0.63
Heroin and fentanyl	153 (57.7)	108 (54.6)	45 (67.2)	0.07
Speedball	38 (14.3)	23 (11.6)	15 (22.4)	**0.03**
Methamphetamine	109 (41.3)	71 (36.0)	38 (56.7)	**0.003**
Methamphetamine and heroin	75 (28.3)	47 (23.7)	28 (41.8)	**0.005**
Prescription opioids	21 (7.9)	17 (8.6)	4 (6.0)	0.49
Tranquilizers	13 (4.9)	9 (4.6)	4 (6.0)	0.65
Buprenorphine	13 (4.9)	9 (4.6)	4 (6.0)	0.64
Suboxone	17 (6.4)	14 (7.1)	3 (4.5)	0.45
Number of drugs injected, *M* (SD)	3.0 (1.8)	2.9 (1.7)	3.6 (2.0)	**0.006**
Acquired sterile syringes from an SSP	225 (85.6)	167 (84.3)	58 (89.2)	0.33
*Drug use changes during COVID*				
Daily injection frequency				
Less than before	38 (14.5)	33 (16.9)	5 (7.5)	**0.02**
Same as before	140 (53.4)	108 (55.4)	32 (47.8)	
More than before	84 (32.1)	54 (27.7)	30 (44.8)	
Used drugs with others (*n* = 14 missing)				
Less than before	100 (39.8)	78 (41.1)	22 (36.1)	0.32
Same as before	123 (49.0)	94 (49.5)	29 (47.5)	
More than before	28 (11.2)	18 (9.5)	10 (16.4)	
Used mostly in private locations	169 (64.5)	122 (62.6)	47 (70.2)	0.26
Avoided the SSP due to COVID fear	26 (9.9)	19 (9.7)	7 (10.6)	0.83
*Past month drug treatment*				
Any treatment, past month	122 (46.0)	96 (48.5)	26 (38.8)	0.17
MOUD treatment, past month	102 (38.9)	82 (41.6)	20 (30.8)	0.12
Non-MOUD treatment, past month	105 (39.9)	82 (41.6)	23 (34.9)	0.33

Bold: *p* < .05

At the bivariate level (Table 1), participants who reported receptive injection equipment sharing were significantly younger than persons who did not (*p* = 0.04). Participants who identified as sexual minorities (*p* = 0.03), as non-Hispanic White (*p* = 0.004), experienced hunger at least weekly (*p* = 0.04), and who engaged in transactional sex (*p* = 0.02) were significantly more likely than their counterparts to report receptive injection equipment sharing. Participants with a high school education were more likely to report receptive injection equipment sharing than participants with other education levels (*p* = 0.01). Use of speedball (*p* = 0.03), methamphetamine (*p* = 0.003), and methamphetamine and heroin (*p* = 0.005) were all significantly associated with receptive injection equipment sharing. Participants who reported receptive injection equipment sharing, on average, used significantly more drugs than persons who did not (*p* = 0.006). Individuals who reported increased injection frequency during COVID-19 were significantly more likely to report receptive injection equipment sharing than persons who reported the same or less frequent injection (*p* = 0.02).

In the multivariable model (Table 2), having a high school education or equivalent was associated with greater odds of receptive injection equipment sharing compared to having less than a high school education (adjusted odds ratio [aOR] = 2.14, 95% Confidence Interval [95% CI] 1.24, 3.69). Experiencing weekly hunger (aOR = 1.89, 95% CI 1.01, 3.56) and number of drugs injected (aOR = 1.15, 95% CI 1.02, 1.30) were also associated with greater odds of receptive injection equipment sharing. Older age (aOR = 0.97, 95% CI 0.94, 1.00) and living in a non-metropolitan area (aOR = 0.43, 95% CI 0.18, 1.02) were marginally associated with decreased odds of receptive injection equipment sharing.

**Table 2:** Multivariable logistic regression results for receptive injection equipment sharing

	Adjusted odds ratio	*p* value	95% CI
Age	0.97	0.08	0.94, 1.00
Male/man gender	0.81	0.40	0.49, 1.33
Sexual minority	1.53	0.40	0.57, 4.12
Transactional sex	1.75	0.23	0.71, 4.35
Racial/ethnic minority	0.74	0.56	0.28, 1.98
Education			
Less than high school	Ref	–	–
High school diploma or equivalent	2.14	0.01	1.24, 3.69
Some college or more	0.88	0.75	0.40, 1.94
Urbanicity			
Large Metropolitan	Ref	–	–
Small Metropolitan	1.11	0.83	0.44, 2.80
Non-Metropolitan	0.43	0.06	0.18, 1.02
Daily injection frequency during COVID			
Less than before	Ref	–	–
About the same	2.08	0.40	0.38, 11.30
More than before	3.37	0.21	0.51, 22.43
Weekly hunger	1.89	0.05	1.01, 3.56
Number of drugs injected	1.15	0.02	1.02, 1.30
Last month drug treatment	0.69	0.22	0.38, 1.25

## Discussion

Using data from a geographically diverse sample of PWID during the early months of the COVID-19 pandemic, we found that approximately one in four participants reported having recently engaged in receptive injection equipment sharing. Factors associated with greater odds of recent receptive injection equipment sharing included experiencing hunger, number of drugs injected, and having a high school diploma. Our findings contribute to existing literature that examines receptive injection equipment sharing by demonstrating that this behavior was associated with factors identified in similar research that occurred before COVID-19 [7, 9, 47, 48]. Eliminating infectious disease transmission among PWID will require novel, low-threshold interventions (e.g., peer-led SSPs, harm reduction vending machines, no-cost access to mail-order harm reduction supplies) that ensure PWID have access to sterile injection equipment during times of co-occurring crises.

We found that 34% of our sample reported experiencing weekly hunger and that hunger was associated with greater odds of receptive injection equipment sharing. These findings parallel similar research conducted among PWID before the COVID-19 pandemic. For example, food insecurity has been associated with PWID engaging in high-risk behaviors (e.g., syringe sharing, condomless sex) for HIV/STI acquisition in prior research [9, 47–49]. For PWID with insufficient food access, obtaining food may compete with persons’ engagement in health-promoting behaviors, such as always using sterile injection equipment. It is also plausible that hunger is a proxy for a mosaic of structural vulnerabilities (e.g., homelessness, unemployment) and having less agency to engage in risk minimizing behaviors. Among PWID living with HIV, research has also shown that inadequate food access increases severity of infectious diseases [50, 51]. Communities should work to guarantee no person struggles with hunger. Strategies to mitigate hunger among PWID, and communities more broadly, should be holistic in nature given the overlapping nature of hunger with other structural vulnerabilities, including homelessness. Comprehensively addressing structural vulnerabilities among PWID may carry significant public health benefits via supporting reductions in high-risk injection behaviors. Future work should be conducted to identify exemplar models of care that integrate the provision of harm reduction services and food access. Notably, there are examples of service providers that integrate food provision and harm reduction [52–54].

Similar to research conducted before COVID-19, we found that the number of drugs PWID injected was positively associated with receptive injection equipment sharing [9]. This finding may be partially explained by associated needs for sterile injection equipment, i.e., persons who inject more types of drugs may require larger volumes of sterile injection equipment, including syringes. Given that the COVID-19 pandemic reduced access to SSPs, it is also plausible that PWID may have had challenges ensuring they had a sterile syringe and other supplies for each injection [55]. Further, many communities lack SSP access, potentially exacerbating risks for receptive injection equipment sharing [26]. Future work should be conducted to develop innovative strategies that afford PWID reliable and low threshold access to sterile injection equipment. Exemplar strategies to increase access to sterile injection equipment may include public health vending machines, mail order injection supplies, and distributing supplies at retail venues (e.g., pharmacies). Peer-based SSPs may also be particularly effective at reaching vulnerable PWID [56, 57].

We found that living in a non-metropolitan area was marginally associated with decreased odds of recent injection equipment sharing. This finding warrants additional study given that many injection drug use-associated HIV outbreaks in rural communities have occurred in recent years [17–19, 58]. Further, analyses that examined risks for injection drug use-associated HIV outbreaks identified many rural counties throughout the United States as vulnerable [59]. Though methodological differences limit comparability across studies (e.g., we recruited PWID who accessed services at drug treatment and harm reduction programs, which may be of limited availability in non-urban areas), receptive injection equipment sharing has been shown to be a relatively common phenomenon among rural PWID [3, 13, 15, 60, 61]. Our finding that non-metropolitan residence was associated with decreased odds of recent injection equipment sharing may also reflect both the considerable heterogeneity in where we recruited participants as well as how we operationalized urbanicity. Nevertheless, future studies should be conducted to more comprehensively understand factors associated with receptive injection equipment sharing among rural PWID and if these relationships are affected by the degree to which persons access drug treatment and harm reduction services.

It is important to interpret the findings of this study relative to its limitations. Our outcome focused on PWID engaging in receptive injection equipment sharing in the past month. As such, we are only able to glean a snapshot of receptive injection equipment sharing among our participants rather than more comprehensive examinations of this behavior and how it may vary by context over time. Additionally, there is considerable variation in how high-risk injection practices are measured in the literature, limiting our ability to make direct comparisons. Due to sample size limitations, we trichotomized our measure of urbanicity. More robust sample sizes may afford nuanced analyses across the urban–rural continuum. In addition, we found that education was significantly associated with receptive injection equipment sharing; however, this finding should be interpreted with caution given both sample size constraints and our sampling strategy. Future lines of scientific inquiry should explore the role of educational attainment and engagement in high-risk injection practices. Efforts should also be undertaken to ensure PWID receive evidence-based education about the risks of sharing injection equipment. Another potential limitation relates to sampling bias given that we recruited persons from substance use disorder and harm reduction service providers in nine states and the District of Columbia. Our findings should not be considered representative of PWID across the US, nor reflective of the experiences of PWID who do not access substance use disorder treatment facilities or harm reduction services. Though our study is not without limitations, it contributes to the public health literature by examining factors associated with receptive injection equipment sharing among a sample of geographically diverse PWID during the early months of a global pandemic.

In conclusion, we found that a quarter of PWID who were connected to drug treatment and harm reduction service providers reported receptive injection equipment sharing during the early months of the global COVID-19 pandemic, and that these behaviors varied according to education level, hunger, urbanicity and number of drugs injected. We also found that PWID residing in non-metropolitan communities had marginally decreased odds of receptive injection equipment sharing. Factors associated with receptive injection equipment sharing in our study had both similarities and differences to prior research. The COVID-19 pandemic affected risks for infectious disease acquisition among PWID throughout the world, and our results shed light on the high-risk injection practices among PWID that contributed to enduring infectious disease risks during the pandemic.

## Data Availability

Deidentified data that supported the findings of this study are available upon reasonable request.

## Abbreviations

SSP: Syringe services programs
PWID: People who inject drugs
HIV: Human immunodeficiency virus
